# Relation of Biomarkers of Inflammation and Oxidative Stress with Hypertension Occurrence in Lone Atrial Fibrillation

**DOI:** 10.1155/2015/653026

**Published:** 2015-07-01

**Authors:** Marija M. Polovina, Miodrag C. Ostojic, Tatjana S. Potpara

**Affiliations:** ^1^Cardiology Clinic, Clinical Center of Serbia, Visegradska 26, 11000 Belgrade, Serbia; ^2^School of Medicine, Belgrade University, Dr. Subotica 8, 11000 Belgrade, Serbia

## Abstract

We compared plasma levels of biomarkers of inflammation (CRP) and oxidation (oxLDL), determined at study inclusion in lone atrial fibrillation (LAF) patients (48.6 ± 11.5 years; 74.0% men) and sinus rhythm controls (49.7 ± 9.3 years; 72.7% men, *P* > 0.05), and investigated the association of baseline CRP and oxLDL levels with the risk for vascular disease (VD) development (hypertension, cerebrovascular disease, coronary/peripheral artery disease, and pulmonary embolism) during prospective follow-up. Baseline CRP (1.2 [0.7–1.9] mg/L versus 1.1 [0.7–1.6] mg/L) and oxLDL levels (66.3 ± 21.2 U/L versus 57.1 ± 14.6 U/L) were higher in LAF patients (both *P* < 0.05). Following a median of 36 months, incident VD occurred in 14 (28.0%) LAF patients, all of whom developed arterial hypertension, and in 5 (11.4%) controls (hypertension, *n* = 4; coronary artery disease, *n* = 1), *P* < 0.05. LAF patients developed VD more frequently and at a younger age. Both CRP (HR, 2.54; 95% CI, 1.26–5.12; *P* = 0.009) and oxLDL (HR, 2.24; 95% CI, 1.14–4.40; *P* = 0.019) were multivariate predictors of incident hypertension in LAF patients, but not in the controls. Further research should clarify clinical relevance of investigated biomarkers for risk stratification and treatment of LAF patients.

## 1. Introduction

Lone atrial fibrillation (AF) is a term commonly used to denote AF occurring in a small subset (~3%) of patients without identifiable cardiovascular and extracardiac comorbidities or triggering factors [1, 2]. With growing understanding of AF pathophysiology, the existence of truly lone AF has been challenged, since emerging risk factors and evidence of subclinical vascular disease have been found in some apparently lone AF patients [2–4]. In particular, latent or masked arterial hypertension has been implicated as a possible “hidden” cause of AF, while clinically overt hypertension has been the most frequently diagnosed vascular disease in cohorts of initially lone AF patients during follow-up [5, 6]. However, determinants of future development of vascular disease in previously lone AF patients have not been broadly investigated.

Current evidence indicates that chronic low-grade inflammation in association with oxidative stress could represent a link between AF and (subclinical) vascular disease [7–10]. Increased plasma levels of inflammatory (e.g., C-reactive protein—CRP) [13] and oxidative mediators (e.g., oxidized low density lipoprotein—oxLDL) have been reported in subjects with lone AF compared to healthy individuals in sinus rhythm [11, 12]. Increased inflammatory and oxidative burden has been associated with AF recurrence and perpetuation [11], but the association of markers of inflammation and oxidative stress with the development of vascular disease in lone AF remains unknown.

The aim of the present study was to evaluate the association of plasma biomarkers of inflammation (CRP) and oxidative stress (oxLDL) with the development of clinically evident vascular disease (arterial hypertension, cerebrovascular disease, coronary/peripheral arterial disease, and pulmonary embolism) in lone AF patients. We hypothesized that if there was a relation between lone AF, inflammation, and oxidative burden, (i) baseline plasma levels of CRP and oxLDL would be higher in lone AF patients compared to healthy controls in sinus rhythm and (ii) CRP and oxLDL could be associated with increased risk for the development of overt vascular disorders in a group of lone AF patients.

## 2. Methods

### 2.1. Study Design and Patient Selection

Of 854 consecutive AF patients referred to the Outpatient Unit of the Cardiology Clinic, Clinical Center of Serbia, between May 2010 and August 2011, we prospectively enrolled 50 subjects (5.8%) with carefully characterized lone AF. We also included a control group of 44 healthy individuals in sinus rhythm, recruited among the hospital staff and acquaintances of AF patients. None of the participants had any evidence of underlying cardiovascular disorders (including hypertension) or extracardiac comorbidities. The study included patients with prior history of AF (paroxysmal, persistent, or permanent), as well as patients with newly diagnosed AF.

### 2.2. Diagnostic Work-Up and Criteria for Comorbidities

Thorough evaluation of medical records, physical examination, office and home blood pressure (BP) measurements, 12-lead electrocardiogram (ECG), laboratory analyses, transthoracic echocardiographic examination, and chest radiography were performed in both AF patients and the controls at inclusion, while additional diagnostic assessments were performed when indicated. Subjects with prior cardiovascular disorders, stroke, transient ischemic attack (TIA), diabetes (fasting plasma glucose ≥7.0 mmol/L or 2 h postload plasma glucose ≥11 mmol/L), hepatic, renal, or thyroid dysfunction, systemic inflammatory disorders, malignancy, or obesity (body mass index [BMI] ≥30 kg/m^2^) were excluded. All participants were required to have normal findings on physical examination, including normal office (systolic BP <140 mmHg and diastolic BP <90 mmHg) and 7-day home BP measurements (systolic BP <135 mmHg and diastolic BP ≥85 mmHg) prior to the institution of any medications. Subjects with high-normal office BP (systolic BP 130–139 mmHg and/or diastolic BP 85–89 mmHg) were included if masked arterial hypertension was excluded by further ambulatory BP monitoring. Arterial hypertension was diagnosed if office BP measurement ≥140/90 mmHg, and/or home BP measurement ≥135/85 mmHg was found (and those subjects were excluded). Normal 12-lead ECG and transthoracic echocardiographic examination, including normal indexes of left atrial volume and left ventricular mass, without evidence of diastolic left ventricular dysfunction or valvular heart disease, were required for all participants. Subjects with symptoms suggestive of obstructive sleep apnea or chronic pulmonary disease were referred for further respiratory function examination. Subjects with symptoms indicative of myocardial ischemia were evaluated by stress-echo exercise testing, supplemented by coronary angiography if indicated. Participants suspected of having lower or upper extremities ischemia or cerebral hypoperfusion were referred for Doppler echosonographic examination to confirm peripheral arterial disease or carotid artery disease, respectively. Patients with a sudden-onset focal neurological deficit suspected of having a stroke or TIA were referred for neurological examination and imaging diagnostics. Peripheral arterial thromboembolism was defined as thromboembolic events outside the brain, retina, heart, or lungs. Pulmonary embolism was suspected in the presence of clinical symptoms, ECG, and laboratory findings and confirmed by computed tomography-pulmonary angiogram.

### 2.3. Biomarker Determination

Fasting peripheral venous blood samples were collected at study inclusion in all participants. Following centrifugation (2,200 rpm, 20 minutes, 4°C), ethylene-diamine-tetra-acetic acid-plasma samples were stored in multiple aliquots at −20°C until further analysis. Plasma oxLDL (Mercodia, Uppsala, Sweden) was determined by commercially available enzyme-linked immunosorbent assay (ELISA). Intra- and interassay coefficients of variation for oxLDL were 6.8% and 7.1%, respectively. Determination of CRP was performed by immunonephelometric technique (Dade Behring BNII Nephelopmeter, Marburg, Germany) with detection limit of 0.2 mg/L and a coefficient of variation of 4.7%.

### 2.4. Follow-Up and Study Outcomes

Follow-up visits were performed by cardiologists at 3-month intervals or more frequently if required, and data on incident vascular disease were collected. A composite study end-point was defined as* the first occurrence of any clinically manifest vascular disease* (arterial hypertension, coronary/peripheral artery disease, significant carotid artery stenosis, pulmonary embolism, stroke, or TIA) in a patient with initially lone AF or in a control group subject. Diagnostic criteria and medical procedures used to confirm the diagnosis of vascular diseases during follow-up were the same as at study inclusion and included assessments of patients' medical records during follow-up, physical exam, 12-lead ECG, and office and 7-day home BP measurements at each visit, supplemented by additional diagnostic tests if indicated (e.g., stress-echo exercise testing, coronary angiography, peripheral/carotid artery Doppler echosonography, CT pulmonary angiography, and endocranial CT scan). Vascular disease was confirmed when standard diagnostic criteria were satisfied. In patients with high-normal office BP at follow-up (systolic BP 130–139 mmHg and/or diastolic BP 85–89 mmHg), ambulatory BP monitoring was performed. Hypertension was diagnosed when either office BP ≥140/90 mmHg and/or home BP ≥135/85 mmHg was found or when 24-hour ambulatory BP of ≥130/80 mmHg was recorded. Control laboratory and echocardiographic exams were performed at 12-month intervals in all participants. Before the development of vascular disease, none of the participants received ACE inhibitors or dihydropyridine Ca^2+^ channel blockers, while statins were prescribed in subjects with elevated plasma cholesterol. In AF patients, vitamin K antagonists were used for the preparation for elective cardioversion. Once vascular disease was diagnosed, patients' treatment was reassessed in accordance with practice guidelines [1]. The study was approved by the local Ethics Committee and all participants gave their informed consent.

### 2.5. Statistical Analysis

Data are presented as mean values and standard deviations for normally distributed continuous variables, medians, and interquartile ranges (25th–75th) for skewed variables and counts (*n*) with percentages (%) for categorical variables. The differences between variables were tested by the two-tailed *t*-test, Mann-Whitney test, *χ* ^2^ test, and Fisher test, respectively. Correlation between normally distributed or log-transformed variables was analyzed by Pearson's correlation.

Univariate Cox proportional hazard analyses were performed to examine the relationships of clinical, echocardiographic, and biochemistry variables from Table 1 with the risk for incident vascular disease. Variables significantly associated with the risk for incident vascular disease were subsequently entered in the multivariate Cox proportional hazard models with plasma biomarkers. The association of plasma biomarkers (CRP and oxLDL) with incident vascular disease was tested in univariate and multivariate Cox proportional hazard models with biomarkers first entered as continuous (oxLDL) or log-transformed (CRP) predictor variables and then as predictor variables categorized per quartile increase. Time to the first occurrence of vascular disease or time to the last follow-up (for those participants who did not develop vascular disease) was used in all Cox proportional hazard analyses.

The *c*-statistic, a measure of the area under the receiver-operator characteristic (ROC) curve, was used to assess the validity of the unadjusted and adjusted models of plasma biomarkers to predict the development of vascular disease during follow-up. Pairwise comparison of the ROC curves was also performed using DeLong approach (the *z*-statistic). A *P* value of <0.05 was accepted as statistically significant. Statistical analyses were performed using SPSS software, version 20 (SPSS, Inc. Chicago, Illinois), or the MedCalc statistical software, version 12.7.0.0.

## 3. Results

### 3.1. Baseline Characteristic of the Study Population

Baseline demographic and clinical characteristics of both study groups are presented in Table 1. Both groups included subjects of similar mean age with male predominance, and there were no differences regarding demographic, clinical, or routine laboratory findings, except for higher BMI and higher levels of fibrinogen in AF patients (Table 1). In AF patients, newly diagnosed AF was noted in 50.0% of subjects. Echocardiographic parameters, including left atrial dimensions and volume index, were similar between AF patients and healthy controls (Table 1). Medical treatment at baseline in AF patients comprised of antiarrhythmic medications and ventricular rate controlling drugs, while none of the healthy controls received any medications (Table 1).

Comparison of baseline plasma levels of the investigated biomarkers revealed that both median CRP level and mean oxLDL level were significantly higher in lone AF patients than in healthy controls (Table 1).

In AF patients, there was a significant positive correlation between plasma levels of CRP and oxLDL (*R* = 0.343, *P* = 0.040), as well as a positive correlation between both biomarkers and patients' age (*R* = 0.520 and *R* = 0.568, resp.). OxLDL was also correlated with total- and LDL-cholesterol concentrations in AF patients (*R* = 0.432 and *R* = 0.460, resp.), all *P* < 0.05. Positive correlations between CRP and oxLDL were also noted in the control group (*R* = 0.334, *P* = 0.028).

### 3.2. Vascular Disease Occurrence during Follow-Up

**Figure 1:**
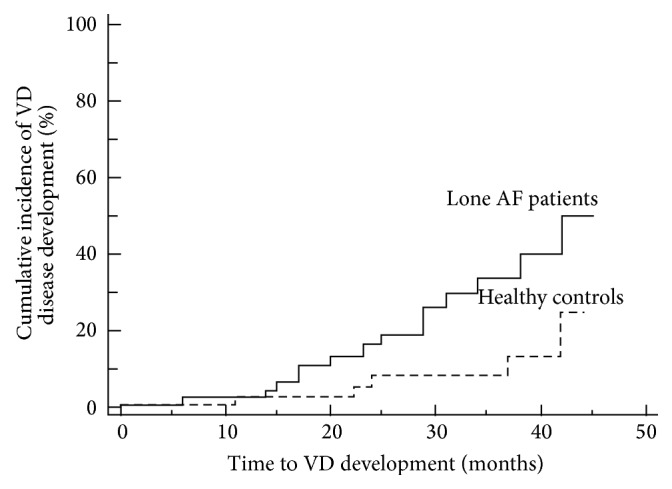
The Kaplan-Meier curve of cumulative risk for vascular disease development in lone AF patients and healthy controls (logrank *P* = 0.014). VD: vascular disease.

In the course of a median 36-month follow-up, incident vascular disease was diagnosed in 14 (28.0%) lone AF patients and in 5 (11.4%) healthy controls (Table 2). Arterial hypertension was the only vascular disorder diagnosed in lone AF patient group. None of the other overt vascular disorders (coronary/peripheral artery disease, significant carotid artery stenosis, pulmonary embolism, stroke, or TIA) occurred in lone AF patients. In the control group, 4 subjects developed arterial hypertension and 1 subject was diagnosed with coronary artery disease. None of the study participants died or were lost to follow-up. The median time to incident vascular disease was similar in both groups, but AF patients developed vascular disease at a younger age compared to healthy controls (Table 2). During follow-up, AF patients were treated with antiarrhythmic medications, beta-blockers, and non-dihydropyridine Ca^2+^ channel blockers, while vitamin K antagonists were prescribed for elective cardioversion (Table 2). One AF patient received aspirin for thromboprophylaxis. Control group patients received no cardiovascular medications except statins (Table 2). The Kaplan-Meier curve of the cumulative risk for vascular disease development in lone AF patients and healthy controls is presented in Figure 1.

### 3.3. Predictors of Vascular Disease Occurrence

In lone AF patient group, subjects with incident hypertension were older, had a longer history of AF, and higher baseline systolic and diastolic BP, BMI, fibrinogen, and total- and LDL-cholesterol plasma levels (Table 3). All these characteristics were significantly associated with the risk of developing hypertension (all *P* < 0.05). There were no differences regarding other clinical, laboratory, or echocardiographic characteristics, including AF type, smoking habits, alcohol intake, or medications used during follow-up (not presented).

**Figure 2:**
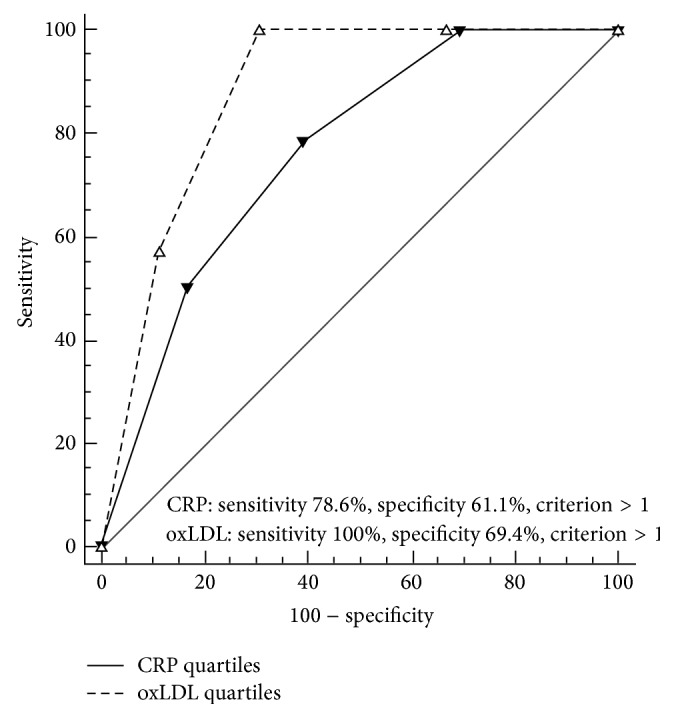
Receiver-operator characteristic (ROC) curves for the predictive validity of unadjusted CRP and oxLDL (per quartile increase) in lone AF patients. 1st, 2nd, 3rd, and 4th quartiles of biomarkers, respectively: CRP: <0.80 mg/L; 0.80 to <1.58 mg/L; 1.58 to <2.70 mg/L; ≥2.70 mg/L; oxLDL: <49.5 U/L; 49.5 to <61.5 U/L; 61.5 to <79.0 U/L; ≥79.0 U/L.

On univariate Cox proportional hazard analyses, both CRP and oxLDL were associated with increased risk for incident hypertension in lone AF patients, both as continuous variables (not presented) and per quartile increase (Table 4). Both biomarkers demonstrated high sensitivity and specificity for incident hypertension (Figure 2) and their predictive ability did not significantly differ between biomarkers when pairwise comparison was performed (Table 4).

On multivariate Cox proportional hazard analysis, investigated biomarkers maintained their predictive significance for subsequent occurrence of hypertension in lone AF patients, with high discriminatory ability that was similar between models in pairwise comparison (Table 5). Multivariate Hazard Ratios (HR) with 95% Confidence Intervals (CI) for CRP and oxLDL were 2.54 (1.26–5.12), *P* = 0.009, and 2.24 (1.14–4.40), *P* = 0.019, respectively.

In the control group, predictors of vascular disease occurrence were older age, higher systolic and diastolic BP, and higher plasma total- and LDL cholesterol levels (Table 3). On univariate Cox regression analysis, there was no statistically significant association of either CRP (HR 1.64, 95% CI, 1.13–7.34, *P* = 0.068) or oxLDL (HR, 1.23, 95% CI, 0.84–6.78, *P* = 0.223) with the risk for incident vascular disease in healthy controls.

## 4. Discussion

In the present study, we have demonstrated increased plasma levels of biomarkers of inflammation (CRP) and oxidative stress (oxLDL) in carefully characterized lone AF patients compared to healthy controls in sinus rhythm. We have also demonstrated that baseline plasma levels of CRP and oxLDL are associated with the risk for incident vascular disease (hypertension) during a median 3-year follow-up in lone AF patients, but not in healthy controls. Despite positive mutual correlations, both CRP and oxLDL were strong multivariate predictors for incident hypertension in our group of lone AF patients. Vascular disease occurred more frequently and at a younger age in lone AF patients than in healthy controls.

Thus far, prognostic significance of biomarkers of inflammation and oxidative burden in lone AF has been demonstrated for arrhythmia perpetuation following cardioversion or catheter ablation [11, 13, 14]. To our knowledge, this is the first study to report on the link between increasing levels of CRP and oxLDL and the development of arterial hypertension in patients with lone AF. From the clinical perspective, development of any vascular disorder, including arterial hypertension, in lone AF patients implies an increased risk of stroke, heart failure, and other complications [1, 5, 15]. Therefore, efforts to establish contributors or associates of vascular disease development in lone AF might aid to earlier recognition and adequate treatment of patients at risk, since it pertains to their prognosis. Such AF patients merit a more intensive follow-up, rigorous life-style changes, and possibly earlier institution of medical treatment to prevent vascular disorders and their complications, while appropriate AF thromboprophylaxis should be commenced once vascular disease becomes apparent.

Although heterogeneous pathophysiological mechanisms may contribute to the development of lone AF, there is a considerable interest in the role of inflammation and oxidative stress as underlying mechanisms. Despite evidence of inflammatory atrial lesions [16] and increased levels of various biomarkers in lone AF [17], there is no unequivocal link between inflammation, oxidative burden, AF, and vascular disease. In the present study, we have demonstrated higher CRP levels in AF patients compared to healthy controls, which is in line with our previous findings of impaired endothelial function and increased CRP concentrations in subjects with persistent lone AF compared to healthy individuals [18]. Similarly, increased levels of CRP [13] and other mediators of inflammation [19] have been reported in other studies of lone AF patients, with a stepwise CRP elevation associated with increased AF burden [13]. However, data on inflammatory mediators in lone AF are still conflicting since some studies have failed to demonstrate higher inflammatory burden in lone AF [20].

We have also found higher oxLDL levels in lone AF patients compared to the controls. Data on oxLDL in lone AF are sparse, but in a recent study, Kim et al. also reported increased plasma concentrations of oxidized lipoproteins in females with paroxysmal lone AF compared to healthy controls [12]. The origin of the observed abnormalities in lone AF remains elusive, but it is conceivable that they represent harbingers of a pathological process that predates an overt vascular disease and first becomes manifest as (lone) AF. The results of the present study support the notion that in the background of some lone AF cases there are inflammatory and oxidative mechanisms (as reflected by increased plasma concentrations of CRP and oxLDL compared to healthy subjects) that are associated with or possibly contributing to the development of vascular disease (e.g., arterial hypertension).

In subjects without AF, several epidemiological studies have established an association of CRP with the occurrence of hypertension [21, 22] while oxLDL has been linked to the development of subclinical vascular disease [23]. Presently, we have not observed a significant relation of either CRP or oxLDL with a risk for incident vascular disease in healthy controls, which does not preclude an association in a larger group of subjects with more pronounced overall cardiovascular risk. Additionally, the absence of an association of the investigated biomarkers with incident vascular disease in the control group further strengthens clinical significance of the association observed in lone AF patients.

Considering other variables associated with hypertension occurrence in lone AF patients in the present study, we have found that aging, longer AF history, higher baseline systolic and diastolic BP, higher serum cholesterol and fibrinogen levels, and increased BMI are linked with incident hypertension. Nevertheless, all of the listed variables were within reference limits acceptable for the healthy population and comparable to the healthy controls, except for higher fibrinogen levels and BMI in AF patients. Longer overall history of AF has been already linked to the risk for vascular disease development in lone AF, possibly due to longer exposition to as yet unrecognized causal or confounding factors [6].

Earlier studies have reported occurrence of arterial hypertension in lone AF between 7.5% (after 7 years) [24] and 44% (after 2 years) [3]. In a recent trial, Weijs et al. reported increased incidence, younger age at onset, and more severe characteristics of vascular disease in lone AF patients compared to healthy subjects [6]. During a 5-year follow-up, incident hypertension occurred in 30% of lone AF patients, while other comorbidities (coronary artery disease, heart failure and stroke) occurred in 49% of patients [6]. Similar to Weijs et al., we have also demonstrated that lone AF patients developed vascular disease (i.e., hypertension) more frequently and at a younger age compared to sinus rhythm controls, but despite careful follow-up we have not documented occurrence of other vascular disorders. However, it is not precluded that over time other comorbidities and vascular complications would become apparent with a possible relation to inflammation and oxidative burden.

The most important limitation to the present study is a relatively small number of highly selected participants in both study groups, originating from a single ethnic background and from one referral center. Therefore, our results do not necessarily apply to all lone AF patients and should be reproduced in other groups of lone AF subjects before any general conclusions could be drawn. Biomarker determination has been conducted at a single time point at study inclusion and we have no information on possible changes in the levels of investigated biomarkers during the follow-up or whether these changes (if occurred) were pertinent to vascular disease development. Furthermore, only 2 highly correlated biomarkers have been evaluated, and adjustments have been conducted for the routine biochemistry parameters and clinical characteristics of study participants, while other possible confounding biochemical variables or risk factors might have been missed. Despite our meticulous efforts to include strictly lone AF patients, some cases with occult comorbidities, particularly latent arterial hypertension, might have been missed. Considering that we have not demonstrated an association of the investigated biomarkers with vascular disease development in the control group, no comparisons of their predictive abilities could have been made between lone AF patients and healthy controls.

## 5. Conclusions

The present study showed that levels of circulating biomarkers of inflammation, CRP, and oxidative stress, oxLDL, are higher in lone AF patients compared to healthy individuals and that both biomarkers were associated with increased risk for incident hypertension in lone AF. Further research should better define pathophysiological role of these biomarkers in the development of AF and vascular disease and refine their clinical relevance in lone AF patients, particularly concerning risk stratification and therapeutic implications. Meanwhile, a regular clinical follow-up of lone AF patients should be routinely conducted in daily clinical practice.

## Figures and Tables

**Table 1:** Baseline characteristics of lone AF patients and healthy controls.

Baseline characteristics	AF patients *n* = 50	SR controls *n* = 44	*P* value

Age (years)	48.6 ± 11.5 (range: 27–65 years)	49.7 ± 9.3 (range: 30–66 years)	0.599
Sex (male)	37 (74.0)	32 (72.7)	0.115
Paroxysmal AF	34 (68.0)	—	—
Newly diagnosed AF	25 (50.0)	—	—
Time since AF diagnosis (months)	36.0 (12.0–108.0)	—	—
Current smokers	9 (18)	10 (22.7)	0.614
Moderate alcohol intake	11 (22.0)	9 (20.4)	0.344
Systolic BP (mmHg)	120.8 ± 10.0	118.5 ± 9.8	0.257
Diastolic BP (mmHg)	75.8 ± 5.5	75.0 ± 7.8	0.593
Heart rate (beats per minute)	64.5 ± 14.3	70.7 ± 7.4	0.106
BMI (kg/m^2^)	25.6 ± 3.5	23.8 ± 2.22	0.011
*Biochemical parameters *			
Fasting blood glucose (mmol/L)	4.8 ± 0.5	4.6 ± 0.5	0.178
Blood urea nitrogen (mmol/L)	5.8 ± 1.2	5.8 ± 1.2	0.788
Serum creatinine (*µ*mol/L)	82.8 ± 15.5	87.4 ± 12.1	0.117
Uric acid (mmol/L)	292.9 ± 67.2	306.7 ± 58.3	0.244
Total cholesterol (mmol/L)	5.7 ± 1.1	5.7 ± 0.8	0.926
HDL-C (mmol/L)	1.1 ± 0.2	1.1 ± 0.3	0.293
LDL-C (mmol/L)	3.8 ± 0.8	3.9 ± 1.0	0.559
Triglycerides (mmol/L)	1.7 ± 0.7	1.8 ± 0.6	0.389
BNP (pg/mL)	68 (51–132)	59 (45–124)	0.788
Fibrinogen (g/L)	3.3 ± 0.6	3.0 ± 0.8	0.018
D-dimer (mg/L)	0.244 (0.193–0.313)	0.235 (0.171–0.298)	0.634
*Plasma biomarkers *			
CRP (mg/L)	1.2 (0.7–1.9)	1.1 (0.7–1.6)	0.030
oxLDL (U/L)	66.3 ± 21.2	57.1 ± 14.6	0.017
*Echocardiographic parameters *			
LA anteroposterior diameter (mm)	39.1 ± 6.2	37.2 ± 5.7	0.114
LA volume index (mL/m^2^)	25.7 ± 6.8	24.2 ± 4.8	0.234
LV end-diastolic dimension (mm)	51.3 ± 4.2	50.8 ± 3.2	0.766
LV end-systolic dimension (mm)	33.0 ± 3.5	31.3 ± 2.9	0.311
LV mass index (g/m^2^)	73.6 ± 13.1	72.8 ± 14.1	0.421
LVEF (%)	63.6 ± 4.9	64.1 ± 4.7	0.544
*Medical therapy *			
Beta-blockers	15 (30.0)	—	—
Non-dihydropyridine Ca^2+^ channel antagonists	3 (6.0)	—	—
Digoxin	2 (4.0)	—	—
Propafenone	20 (40.0)	—	—
Sotalol	5 (10.0)	—	—
Amiodarone	18 (36.0)	—	—

Data are presented as *n* (%), mean ± standard deviation, or median with interquartile range; AF: atrial fibrillation; BP: blood pressure; BMI: body mass index; HDL-C: high density lipoprotein cholesterol; LDL-C: low density lipoprotein cholesterol; BNP: B-type natriuretic peptide; CRP: C-reactive protein; oxLDL: oxidized low density lipoprotein; LA: left atrial; LV: left ventricular; LVEF: left ventricular ejection fraction.

**Table 2:** Development of vascular disease in lone AF patients and healthy controls.

	AF patients *n* = 50	SR controls *n* = 44	*P* value
VD development	14 (28.0)	5 (11.4)	0.045
Time to VD development (months)	28 (9–42)	34 (17.5–39.5)	0.391
Age at VD development	56.8 ± 8.3	61.8 ± 3.7	0.031
*Medical therapy prior to VD development *			
Beta-blockers	20 (40.0)	—	—
Non-dihydropyridine Ca^2+^ channel antagonists	2 (4.0)	—	—
Digoxin	0 (0.0)	—	—
Propafenone	14 (28.0)	—	—
Sotalol	1 (2.0)	—	—
Amiodarone	20 (40.0)	—	—
Vitamin K antagonists	6 (12.0)	—	—
Aspirin	1 (2.0)	—	—
Statins	3 (6.0)	4 (9.1)	0.414

AF: atrial fibrillation; SR: sinus rhythm; VD: vascular disease.

**Table 3:** Clinical and laboratory variables associated with vascular disease occurrence.

Clinical characteristics	AF patients, *n* = 50				SR controls, *n* = 44			
	Incident VD (−) *n* = 36 (72.0)	Incident VD (+) *n* = 14 (28.0)	HR (95% CI)	*P* value	Incident VD (−) *n* = 39 (88.6)	Incident VD (+) *n* = 5 (11.4)	HR (95% CI)	*P* value
Age at inclusion (years)	46.2 ± 12.1	54.7 ± 7.3	1.15 (1.05–1.38)	0.013	46.3 ± 7.8	58.6 ± 4.7	1.25 (1.13–4.88)	0.006
Time since AF diagnosis (months)	24 (12–102)	60 (24–108)	1.14 (1.04–2.34)	0.019	—	—	—	—
Systolic BP at inclusion (mmHg)	117.7 ± 9.1	128.6 ± 7.6	1.10 (1.04–1.17)	0.002	114.6 ± 7.9	127.6 ± 7.8	1.50 (1.12–2.78)	>0.001
Diastolic BP at inclusion (mmHg)	74.7 ± 5.8	78.9 ± 3.5	1.16 (1.03–1.32)	0.018	73.6 ± 7.8	80.1 ± 3.5	1.78 (1.23–3.76)	>0.001
BMI (kg/m^2^)	24.8 ± 3.4	26.8 ± 2.9	1.17 (1.01–1.37)	0.040	22.6 ± 2.5	23.8 ± 1.9	1.15 (0.89–1.78)	0.116
Fibrinogen (g/L)	3.2 ± 1.0	3.7 ± 0.8	1.59 (1.13–2.53)	0.043	2.9 ± 1.0	3.1 ± 0.7	1.13 (0.95–1.89)	0.221
Total cholesterol (mmol/L)	3.6 ± 0.8	4.4 ± 0.8	1.99 (1.67–3.38)	0.011	3.8 ± 1.4	4.7 ± 1.3	2.11 (1.55–4.67)	0.001
LDL-C (mmol/L)	3.2 ± 0.5	3.8 ± 0.6	2.28 (1.23–4.21)	0.008	3.0 ± 0.8	3.9 ± 1.2	2.15 (1.66–3.78)	0.004

AF: atrial fibrillation; SR: sinus rhythm; HR: hazard ratio; CI: confidence interval; BP: blood pressure; BMI: body mass index; LDL-C: low density lipoprotein cholesterol.

**Table 4:** Univariate Cox proportional hazard analysis for the association of biomarkers with the occurrence of hypertension in lone AF patients.

Biomarkers	Unadjusted Cox analysis			Biomarker predictive ability (per quartile increase)			Pairwise comparison of biomarker predictive ability (per quartile increase)		
	HR	95% CI	*P* value	*c*-statistic	95% CI	*P* value	*z*-statistic	95% CI	*P* value
CRP (quartiles)^*∗*^	2.35	(1.25–4.39)	0.008	0.763	0.627–0.899	0.004	1.62 (CRP versus oxLDL)	−0.02–0.23	0.1005
oxLDL (quartiles)^*∗*^	2.55	(1.33–4.89)	0.005	0.879	0.787–0.971	<0.001			

HR: hazard ratio; CI: confidence interval.

^*∗*^1st, 2nd, 3rd, and 4th quartiles of biomarkers, respectively: CRP: <0.80 mg/L; 0.80 to <1.58 mg/L; 1.58 to <2.70 mg/L; ≥2.70 mg/L; oxLDL: <49.5 U/L; 49.5 to <61.5 U/L; 61.5 to <79.0 U/L; ≥79.0 U/L.

**Table 5:** Multivariate Cox proportional hazard models for the association of biomarkers with the occurrence of hypertension in lone AF patients.

Biomarkers	Multivariable Cox analysis			Model discriminatory ability			Pairwise comparison of model discriminatory ability		
	HR	95% CI	*P* value	*c*-statistic	95% CI	*P* value	*z*-statistic	95% CI	*P* value
CRP (per quartile increase)	2.54	1.26–5.12	0.009	0.905	0.819–0.990	<0.001	0.322 (CRP versus oxLDL)	−0.073–0.101	0.7477
oxLDL (per quartile increase)	2.24	1.14–4.40	0.019	0.925	0.850–0.999	<0.001			

HR: hazard ratio; CI: confidence interval.

## References

[1] Camm A. J., Kirchhof P., Lip G. Y. (2010). Guidelines for the management of atrial fibrillation: the Task Force for the Management of Atrial Fibrillation of the European Society of Cardiology (ESC). *Europace*.

[2] Potpara T. S., Lip G. Y. H. (2014). Lone atrial fibrillation—an overview. *International Journal of Clinical Practice*.

[3] Katritsis D. G., Toumpoulis I. K., Giazitzoglou E. (2005). Latent arterial hypertension in apparently lone atrial fibrillation. *Journal of Interventional Cardiac Electrophysiology*.

[4] Weijs B., Pisters R., Haest R. J. (2012). Patients originally diagnosed with idiopathic atrial fibrillation more often suffer from insidious coronary artery disease compared to healthy sinus rhythm controls. *Heart Rhythm*.

[5] Potpara T. S., Stankovic G. R., Beleslin B. D. (2012). A 12-year follow-up study of patients with newly diagnosed lone atrial fibrillation. Implications of arrhythmia progression on prognosis: the Belgrade atrial fibrillation study. *Chest*.

[6] Weijs B., de Vos C. B., Tieleman R. G. (2013). The occurrence of cardiovascular disease during 5-year follow-up in patients with idiopathic atrial fibrillation. *Europace*.

[7] Sung K. C., Suh J. Y., Kim B. S. (2003). High sensitivity C-reactive protein as an independent risk factor for essential hypertension. *American Journal of Hypertension*.

[8] Tsioufis C., Syrseloudis D., Hatziyianni A. (2010). Relationships of CRP and P wave dispersion with atrial fibrillation in hypertensive subjects. *American Journal of Hypertension*.

[9] Friedrichs K., Baldus S., Klinke A. (2012). Fibrosis in atrial fibrillation—role of reactive species and MPO. *Frontiers in Physiology*.

[10] Schnabel R. B., Wild P. S., Wilde S. (2014). Multiple biomarkers and atrial fibrillation in the general population. *PLoS ONE*.

[11] Leftheriotis D. I., Fountoulaki K. T., Flevari P. G. (2009). The predictive value of inflammatory and oxidative markers following the successful cardioversion of persistent lone atrial fibrillation. *International Journal of Cardiology*.

[12] Kim S.-M., Lee J.-H., Kim J.-R., Shin D.-G., Lee S.-H., Cho K.-H. (2011). Female patients with atrial fibrillation have increased oxidized and glycated lipoprotein properties and lower apolipoprotein A-I expression in HDL. *International Journal of Molecular Medicine*.

[13] Chung M. K., Martin D. O., Sprecher D. (2001). C-reactive protein elevation in patients with atrial arrhythmias: inflammatory mechanisms and persistence of atrial fibrillation. *Circulation*.

[14] Richter B., Gwechenberger M., Socas A. (2012). Markers of oxidative stress after ablation of atrial fibrillation are associated with inflammation, delivered radiofrequency energy and early recurrence of atrial fibrillation. *Clinical Research in Cardiology*.

[15] Potpara T. S., Polovina M. M., Licina M. M., Marinkovic J. M., Prostran M. S., Lip G. Y. H. (2012). Reliable identification of ‘truly low’ thromboembolic risk in patients initially diagnosed with ‘lone’ atrial fibrillation: the belgrade atrial fibrillation study. *Circulation: Arrhythmia and Electrophysiology*.

[16] Stiles M. K., John B., Wong C. X. (2009). Paroxysmal lone atrial fibrillation is associated with an abnormal atrial substrate: characterizing the ‘second factor’. *Journal of the American College of Cardiology*.

[17] Vílchez J. A., Roldán V., Hernández-Romero D., Valdés M., Lip G. Y., Marín F. (2014). Biomarkers in atrial fibrillation: an overview. *International Journal of Clinical Practice*.

[18] Polovina M., Potpara T., Giga V., Stepanović J., Ostojić M. (2013). Impaired endothelial function in lone atrial fibrillation. *Vojnosanitetski Pregled*.

[19] Li J., Solus J., Chen Q. (2010). Role of inflammation and oxidative stress in atrial fibrillation. *Heart Rhythm*.

[20] Ellinor P. T., Low A., Patton K. K., Shea M. A., MacRae C. A. (2006). C-reactive protein in lone atrial fibrillation. *American Journal of Cardiology*.

[21] Sesso H. D., Buring J. E., Rifai N., Blake G. J., Gaziano J. M., Ridker P. M. (2003). C-reactive protein and the risk of developing hypertension. *The Journal of the American Medical Association*.

[22] Lakoski S. G., Cushman M., Siscovick D. S. (2011). The relationship between inflammation, obesity and risk for hypertension in the Multi-Ethnic Study of Atherosclerosis (MESA). *Journal of Human Hypertension*.

[23] Hulthe J., Fagerberg B. (2002). Circulating oxidized LDL is associated with subclinical atherosclerosis development and inflammatory cytokines (AIR study). *Arteriosclerosis, Thrombosis, and Vascular Biology*.

[24] Rostagno C., Bacci F., Martelli M., Naldoni A., Bertini G., Gensini G. (1995). Clinical course of lone atrial fibrillation since first symptomatic arrhythmic episode. *The American Journal of Cardiology*.

